# Preventing the Obesity Epidemic by Second Generation Tailored Health Communication: An Interdisciplinary Review

**DOI:** 10.2196/jmir.1409

**Published:** 2010-06-28

**Authors:** Heidi Päivyt Karoliina Enwald, Maija-Leena Aulikki Huotari

**Affiliations:** ^1^Finnish, Information Studies and LogopedicsCentre of Excellence in Research, Faculty of HumanitiesUniversity of OuluOuluFinland

**Keywords:** Health communication, health promotion, intervention studies, tailored interventions, tailoring, computer-based delivery, Internet, health behavior change, obesity, public health

## Abstract

**Background:**

The prevention of obesity and health concerns related to obesity are major challenges worldwide. The use of eHealth communication and the tailoring of information delivered via the Internet at the individual level may increase the effectiveness of interventions. Mastering behaviors related to nutrition, physical activity, and weight management are the main issues in preventing obesity, and the need for interdisciplinary knowledge within this area is obvious.

**Objective:**

The objectives were to review the literature on tailored health communication and to present an interdisciplinary analysis of studies on “second” generation tailored interventions aimed at behavior change in nutrition, physical activity, or weight management.

**Methods:**

A literature search was conducted of the main electronic information sources on health communication. Selection criteria were defined, and 23 intervention studies were selected. The content analysis focused on the following: study designs, objectives of behavior change, target groups, sample sizes, study lengths, attrition rates, theories applied, intervention designs, computer-based channels used, statistically significant outcomes from the perspective of tailoring, and possible biases of the studies. However, this was not a structured meta-analysis and cannot be replicated as such.

**Results:**

Of the 23 studies, 21 were randomized controlled trials, and all focused on behavior change: 10 studies focused on behavior change in nutrition, 7 on physical activity, 2 on nutrition and physical activity, and 4 on weight management. The target groups and the number of participants varied: 8 studies included more than 500 participants, and 6 studies included less than 100. Most studies were short; the duration of 20 studies was 6 months or less. The Transtheoretical Model was applied in 14 of the 23 studies, and feedback as a tailoring mechanism was used in addition to an Internet site (or program) in 15 studies and in addition to email in 11 studies. Self-reporting was used in 15 studies, and 14 studies did not have a no-information control group. Tailoring was more effective in nutrition interventions than in physical activity and weight management interventions. The outcomes were mixed or negative in 4 studies of physical activity interventions and in 3 studies of weight management. The use of a no-information control group seemed to have been linked to statistically significant between-group effects in measuring physical activity. This bias effect related to intervention design may explain the differences in the outcomes of the physical activity studies.

**Conclusions:**

Tailoring was shown to have been an effective method in nutrition interventions, but the results for physical activity were mixed, which is in line with previous studies. Nevertheless, the effect of possible biases, such as relying solely on self-reports and on intervention design without a no-information control group, should not be underestimated. Thus, the issue of bias merits more attention in planning interventions and in future meta-analyses.

## Introduction

Obesity and overweight, which are associated with the metabolic syndrome, type 2 diabetes, and heart disease, are obvious health problems in Western countries and are also increasing in Asia and Africa. Health communication is a key strategy in informing the public about health concerns [1], but conventional approaches are the least effective as they consist of passive dissemination of messages from experts to the public in the hope of motivating people to change their behaviors [2]. As the health information environment has changed dramatically during the past decade, partly due to the rapid diffusion of Internet technology [3,4], eHealth communication provides a new means to prevent obesity from becoming a global epidemic. Through unique features such as mass customization, interactivity, and convenience, eHealth may influence the psychosocial factors of control, motivation, and self-efficacy [2], that is, individuals’ expectations about whether they will be able to master a behavior, and if so, how successful they will be [5].

### Targeting and Tailoring Health Communication

We claim that eHealth communication as such is not enough for behavior change at the individual level; it also requires targeting and tailoring information. These strategies combine the benefits of interpersonal communication and mass media and are based on the ideas of social marketing [6]. In targeted communication, the aim is to reach particular population subgroups whose members share the same characteristics. In tailored communication, the aim is to reach specific individuals [7] through three mechanisms: personalization, feedback, and adaptation (ie, content matching). These tailoring mechanisms tend to be used in combination (see, for example, [8-11]).

Research indicates that tailored health communication may be more effective than traditional promotion [2,12-14]. Tailored health communication is seen as more satisfying and personally relevant, being read more thoroughly, and discussed with others more often [7,12,13,15]. Tailoring may enhance the motivation for processing health information in at least four ways: (1) by matching content to an individual’s information needs and interests, (2) by framing health information in a context that is meaningful to the person, (3) by using design and production elements to gain the individual’s attention, and (4) by providing the quality and quantity of information desired and through channels of delivery preferred by the individual, thereby potentially reducing barriers to exposure to interventions [7,16].

The outcomes of tailored health communication can be assessed by studying a specific intervention in which behavioral, physiological, and/or psychological factors are measured at baseline and at the end of the intervention or follow-up, and the results are compared. In addition to determining whether the tailoring element has been effective, the tailored group needs to be compared with the control group, which is a group provided with general information or no information. However, the intervention designs may differ greatly, and the outcomes and effectiveness can be measured and estimated in various ways, complicating evaluation and comparison of the intervention studies reported in the research literature. This justifies examination of specific details, such as the target audience or the length of the intervention period [17], to understand how interventions are built.

Delivery of computer-generated tailored information may differ from print (eg, [18]), telephone [19]), mobile phone (eg, [20]), CD-ROM (eg, [21]) or the Internet (eg, [22]). Computer-tailored but print-delivered interventions, for example, computer-generated printed pamphlets, are deemed the “first” generation, and interventions using interactive media are deemed the “second” generation of tailored health communication [23]. The “third” generation interventions refer to interventions delivered via mobile and remote devices such as mobile phones and handheld computers [24]. In this paper we focus on intervention studies utilizing second generation tailored health communication.

### Theories Applied in Second Generation Tailored Health Communication

Improved theoretical understanding in building interventions may enhance their outcomes. The theoretical basis of tailored communications derives from social psychology and communication and persuasion theories and models [25]. The construction of interventions to change beliefs toward behavior may be based on behavior change theories [26] as well as information processing theories. Also, consideration of whether the message content has been tailored for different audiences may help explain its effectiveness or ineffectiveness in changing behavior [26].

Tailored feedback may be based on social psychological theories, for example, the Health Belief Model (HBM) by Rosenstock [27] and Becker [28] or the Transtheoretical Model (TTM) by Prochaska and DiClemente [29]. The HBM predicts that individuals are more likely to act and change their health behavior when at risk and when the perceived benefits of taking action outweigh the perceived costs or barriers. The TTM claims that individuals move through a series of five stages of change in the adoption of healthy behaviors or cessation of unhealthy ones. The TTM is most often used in tailored health interventions [30]. The Precaution Adoption Process Model (PAPM) by Weinstein [31] is another stage-based model. This model describes how a person decides to take action as well as how a person translates that decision into action.

The Elaboration Likelihood Model (ELM) of persuasion by Petty and Cacioppo [32] is based on the assumption that under many circumstances people are active information processors who “think about messages carefully, relate them to other information they have encountered in the past, and consider the messages in the context of their own life experience” [33]. This suggests that people are more likely to process information thoughtfully if they perceive it to be personally relevant. The ELM also distinguishes between central and peripheral routes to persuasion.

Bandura’s [34] Social Cognitive Theory (SCT) permits the assumption that messages can be tailored according to different levels of self-efficacy. The Health Promotion Model (HPM) by Pender [35] is also connected to the SCT. Studies have indicated that using the Internet in tailored SCT interventions have achieved changes in nutrition practices, physical activity, and weight loss, and that the participants have maintained these changes for up to a year [36].

Other theories underlying second generation tailored health communication include the Theory of Reasoned Action (TRA) and the Theory of Planned Behavior (TPB). These theories posit that the most proximal predictor is behavioral intention, or the perceived likelihood of performing behavior [37]. Webb et al [38] suggested that the effectiveness of second generation interventions is associated with more extensive use of theory in general and with the TPB in particular. Another such theory is the Goal Setting Theory (GST). The idea behind the GST is that setting goals specifies the objectives of behavior, directs effort to goal-relevant activities, and increases commitment [39].

Combining demographic and/or behavior concepts with the theoretical frameworks of tailoring has been shown to be efficacious in interventions [37]. We can also claim that careful tailoring on demographic characteristics (eg, gender, race, and age) and feedback provided on the behavior itself may enhance the effectiveness of theoretical tailoring. (See also [40].)

### Examples of Meta-analyses of First, Second, and Third Generation Health Interventions

To the best of our knowledge, this paper is among the first interdisciplinary reviews within the context of second generation computer-tailored health interventions. The foci of many meta-analytic reviews of general Internet-based health behavior change interventions have included nutrition, physical activity, and weight management as well as other health behaviors. Meta-analyses of Internet-based physical activity interventions have been conducted by van den Berg et al [41] and Marcus et al [42], for example. A meta-analysis by Wantland et al [43] compared (tailored or nontailored) second generation and other types of health interventions. In this meta-analysis [43], most of the studies revealed improved knowledge and/or improved behavioral outcomes for participants involved in second generation interventions. In another meta-analysis, Norman et al [24] studied eHealth interventions for physical activity and dietary behavior change.

Meta-analytic reviews of first and second generation interventions are provided, for example, by Kroeze et al [30], who scrutinized computer-tailored interventions on physical activity and nutrition education. This group of authors found that 3 of the 11 physical activity studies and 20 of the 26 nutrition studies showed significant effects of the tailored interventions, and the evidence was most consistent for tailored interventions on fat reduction [30]. Neville et al [44], in their analysis of second and third generation interventions, focused on dietary behavioral change and found that 8 of 12 interventions had significant positive effects on dietary behavior [44].

We found only one meta-analysis on second generation tailored interventions related to nutrition, physical activity, and weight management. This review, by Lustria et al [8], screened over 500 studies and selected 30 for the analysis to ascertain how these interventions were implemented and delivered via the Internet and what mechanisms and criteria were used to individualize health messages [8]. The selected interventions spanned four broad areas (nutrition and diet, physical activity, alcoholism, and smoking cessation) and differences in the level of sophistication of message tailoring were identified [8]. Neville et al [45] conducted a systematic review of second and third generation physical activity interventions targeting adults. According to these authors, the evidence of the effectiveness of these interventions was inconclusive.

### Aim of the Study

In this paper, we aimed at presenting an interdisciplinary review of the research literature on health communication to prevent obesity and related health problems, such as metabolic syndrome and type 2 diabetes, at the individual level. We assumed that to succeed in preventing these diseases, it is crucial to master behavior related to nutrition, physical activity, and weight management. We reviewed second generation intervention studies conducted in these three areas of activity by examining specific issues related to the selected interventions. We also compared the studies and their outcomes to identify possible differences and reasons for these.

## Methods

### Search of the Research Literature

The literature searches were performed between January and August 2009. Research literature on health communication and tailoring was sought from the following databases: Pubmed and Ovid (MEDLINE), Science Direct (Elsevier), Google Scholar, Library and Information Science Abstracts (LISA) (CSA), Academic Search Premier (EBSCO), Library, Information Science & Technology Abstracts (LISTA) (EBSCO), Emerald Journals (Emerald), Educational Resources Information Center database (ERIC) (CSA), Scopus, Sociological Abstracts (CSA), Web of Science (ISI), and ABI/Inform (ProQuest). The search terms were: health, health communication, tailor*, Internet, WWW, web, net, online, nutrition, diet*, vegetable/fruit consumption/intake, fat intake, weight, weight management, obesity, overweight and physical activity or exercise. (An asterisk was used to include all terms that began with a particular spelling, such that “diet*” would include dietary and dieting, for example.) The Boolean search queries were based on the following formulations: (tailor* [Title/Abstract/Keywords]) AND (weight OR “weight management” OR obesity OR overweight OR “physical activity” OR exercise OR “fat intake” OR nutrition OR diet* OR “vegetable consumption/intake” OR “fruit consumption/intake” [Title/Abstract/Keywords]) AND (Internet OR WWW OR web OR net OR online [Title/Abstract/Keywords]).

The searches were not limited by publication date, but the availability of articles was taken into account. So-called pearl-fishing, or chaining, strategy was also used by taking a closer look at the articles cited in other articles and at recent articles citing certain older relevant articles. Many of the articles retrieved were published in high quality, peer-reviewed, international journals of psychology, health promotion, health education, nutrition, medicine, nursing and communication.

### Inclusion and Exclusion Criteria for the Intervention Studies

In order to find examples of intervention studies for the content analysis, articles were included if they: (1) focused on second generation interventions; (2) focused on health behavior related to nutrition, physical activity, or weight management, alone or in combination; (3) measured or assessed behavioral, psychological, or physiological outcomes; (4) were randomized controlled trials or quasi-experimental designs with pretest and posttest; and (5) were available in full text.

Articles were excluded if they: (1) measured only the feasibility and acceptability of computer-delivered tailored health communication, as for example, the studies by Vandelanotte et al [46], Spittaels et al [47], Comrie et al [48], and Maes et al [49]; (2) focused on diabetes self-management, such as the studies by Glasgow et al [50] and Wangberg [51]; or (3) gave advice in computer kiosk or in an online Internet shopping site, such as the study by Huang et al [52].

Finally, 23 articles that clearly met the criteria were selected for the content analysis [15,21-23,53-72] and were analyzed by categorizing them according to the themes of the research questions formulated as follows:

1. What is the study design and setting?
2. Which objectives are set for the behavior change in the selected intervention studies?
3. Who are the target groups?
4. What are the sample sizes?
5. What are the lengths of the studies (follow-up) and what is their attrition rate?
6. On which theories or theoretical concepts is the background of the intervention studies built?
7. What is the intervention design of the studies?
8. What tailoring mechanisms are used?
9. Which Internet-based channels are used to deliver tailored health information?
10. What are the main outcomes of the interventions from the perspective of tailoring?
11. What kind of biases can be identified in the selected studies?

In this paper we use the term “study” to refer to the intervention and its follow-up examined in the articles selected for analysis.

## Results

### Study Design, Objectives, Target Groups, Sample Sizes, Lengths of Follow-up, and Attrition Rates

The study design provides the basis for an intervention study. Of the 23 studies selected, 21 were randomized controlled trials. Only 2 studies used quasi-experimental designs, that is, these were nonrandomized controlled trials [53,55]. In the study by Frenn et al [55], participants were assigned to intervention or control group according to their classroom assignment, and in the study by Block et al [53], participants chose their preferred dietary emphasis for a 12-week program. In 20 of the 23 studies, the intervention was performed in a real-life setting, such as at home. Of the 23 studies, 3 were conducted in a controlled situation [23,55,61] in which the participants performed the assessments and received the tailored information or feedback in classrooms or offices.

The objectives of selected interventions may be important factors for preventing metabolic syndrome, obesity, and type 2 diabetes. The analysis showed that these studies may have concentrated on a single facet of health behavior, such as physical activity, or have tried to influence more than one health behavior. For instance, we found that combining fruit and vegetable consumption and fat intake in the same study was quite common [15,53,57]. Of the 23 studies selected, 10 focused on behavior change in nutrition, 7 on change in physical activity, 2 on change in both nutrition and physical activity, and 4 on behavior change related to weight management. Objectives, target groups, sample sizes, lengths of the studies, and attrition rates are summarized in Table 1.

**Table 1:** Objectives of behavior change, target groups, sample sizes, lengths of follow-up periods, and attrition rates of the selected intervention studies

Author(s) and Year of Publication (n = 23)	Study Focus	Objectives of Behavior Change (Measurement Method)	Target Group	Sample Size	Length of the Study Follow- up in Months	Percent Attrition at Follow-up

Block et al, 2004 [53]	Nutrition	Fruit and vegetable consumption, fat intake, determinants of fruit and vegetable consumption and fat intake (self-report)	Adults	84	3	44
de Vet et al, 2008 [68]	Nutrition	Fruit and vegetable consumption (self-report)	Adults	775	Baseline + 1 week	18
Di Noia et al, 2008 [61]	Nutrition	Fruit and vegetable consumption, determinants of fruit and vegetable consumption (self-report)	Adolescents, minority	549	1	8
Irvine et al, 2004 [57]	Nutrition	Fruit and vegetable consumption, fat intake, determinants of dietary intake (self-report)	Healthy adults	517	2	10
Kroeze et al, 2008 [21]	Nutrition	Fat intake, dietary intake (self-report)	Healthy adults	442	6	13
Luszczynska et al, 2007 [58]	Nutrition	Fruit and vegetable consumption, determinants of fruit and vegetable consumption (self-report)	Healthy adults	285	6	30
Oenema et al, 2001 [23]	Nutrition	Determinants of fruit and vegetable consumption and fat intake (self-report)	Adults	204	Baseline	Immediately posttest
Oenema et al, 2005 [15]	Nutrition	Fruit and vegetable consumption, fat intake, determinants of fruit and vegetable consumption and fat intake (self-report)	Healthy adults	782	1	21
Papadaki and Scott, 2008 [62]	Nutrition	Mediterranean diet score, Fruit and vegetable consumption (self-report) blood lipids (objectively measured)	Women	72	9	27
Park et al, 2008 [63]	Nutrition	Determinants of fruit and vegetable consumption (self-report)	Young adults	160	1	14
Dunton and Robertson, 2008 [54]	Physical activity	Physical activity, determinants of physical activity (self-report)	Women, minority	156	3	29
Hageman et al, 2005 [56]	Physical activity	Physical activity (self report) cardiovascular fitness, % body fat, weight, flexibility (objectively measured)	Older women	31	3	3
Marcus et al, 2007 [59]	Physical activity	Physical activity (self-report), cardiovascular fitness (objectively measured)	Sedentary adults	249	12	12
Napolitano et al, 2003 [60]	Physical activity	Physical activity (self-report)	Sedentary adults	65	3	20
Spittaels et al, 2007 [65]	Physical activity	Physical activity (self-report) weight, blood pressure, % body fat (objectively measured)	Healthy adults	526	6	29
Spittaels et al, 2007 [72]	Physical activity	Physical activity (self-report)	Healthy adults	434	6	34
Wanner et al, 2009 [70]	Physical activity	Physical activity (objectively measured and self-report), determinants of physical activity (self-report)	Adults	1531	13	50
Frenn et al, 2005 [55]	Nutrition and physical activity	Fat intake, physical activity (self-report)	Adolescents, minority	178	1	23
Oenema et al, 2008 [71]	Nutrition and physical activity	Fat intake, physical activity (self-report)	Adults	2159	1	19
Booth et al, 2008 [22]	Weight management	Weight, waist circumference (objectively measured), dietary intake, physical activity (self-report)	Overweight or obese adults	73	3	27
Rothert et al, 2006 [64]	Weight management	Weight (self-report)	Overweight or obese adults	2862	6	80
Tate et al, 2001 [67]	Weight management	Weight, waist circumference (objectively measured), fat intake, dietary intake (self-report)	Overweight or obese adults	91	6	22
Tate et al, 2006 [66]	Weight management	Weight (objectively measured) dietary intake, fat intake, physical activity (self-report)	Overweight or obese adults	192	6	20

Possible changes in health behavior can be monitored by self-reported indicators or by objective physiological measures conducted in controlled conditions. In 15 of the 23 studies, the measures were only self-reported. Objectively measured factors included weight, physical activity, blood pressure, body fat percentage, blood lipids (eg, cholesterol), waist circumference, flexibility, and cardiorespiratory fitness (eg, maximal oxygen uptake [VO2max]). Of these factors, physical activity and weight were self-reported in 13 of the studies.

The studies selected had many kinds of target groups, whose inclusion criteria were, for example, based on age (eg, adolescents) or gender. The choice of women as a target group was explained as follows: “[W]omen were recruited because they are more likely than men to use the Internet for health information and more likely to be responsible for meal planning and preparation” [62].

Of the 23 studies, 3 concentrated on minority groups. The target groups were economically disadvantaged 11 to 14 year-old urban African-Americans [61], low-income culturally diverse seventh grade students [55], and ethnically diverse women [54]. Risk groups also were chosen as targets: sedentary adults were the focus in 2 studies, overweight or obese individuals were the focus in 4 studies. For example, in 1 study, individuals were included who had a body mass index in the range 27 to 40 kg/m² [64]. The selection criteria were also quite strict in some cases. For example, studies may have included only individuals with high BMI and excluded individuals less than 18 years of age, women who were pregnant, or individuals who were taking medication for diabetes [22].

There were large differences in the sample sizes of the studies. Of the 23 included studies, 8 had enrolled more than 500 participants at baseline. On the other hand, in 6 studies the sample sizes were less than 100.

Length of follow-up varied depending on the purpose of the study. Some studies focused on examining short-term effects, such as the immediate impact of Web-based computer-tailored nutrition education on personal awareness and intentions related to intake of fat and fruits and vegetables [23]. Some studies, in turn, tried to ascertain the long-term effects of tailored health communication (eg, 12 months [59] and 13 months [70]). In 20 of the 23 studies, the length of the study or the follow-up period was 6 months or less, and the final measures and observations were made immediately after the participants had received the last intervention contact or some time thereafter. In some of the studies, the attrition rate was decidedly high, but in 18 of 23 studies the attrition rate was under 30%.

### Theories Applied, Intervention Design, Tailoring Mechanisms, and Outcomes

In many of the interventions selected, the assessments and information given to participants were based on theories of behavior change or information processing. The TTM and stages of change and the concept of self-efficacy (SE), which is connected to several theories, such as the SCT and HPM, were mentioned most often in the intervention studies selected. The TTM, including the stages of change, was the most commonly mentioned theory, cited in 14 of the 23 studies. Multiple interventions gave participants stage-tailored information (eg, [55,57,61,63,65,68]), and many measured the stage of change at the beginning and monitored any possible improvement (eg, [22,53,54,60,70]). Other theories or models mentioned in the studies were the ELM [15], PAPM [15,23,71], GST [22], TPB [65], TRA [57], and HPM [56]. Some other theoretical concepts were also mentioned, for example, motivation, awareness of risk behavior, goals and intentions. These are not presented here in detail. In 4 studies [59,62,66,67], no theories were mentioned.

Table 2 presents the theories or theoretical concepts applied or mentioned in the studies selected, use of computer for delivering tailored information, intervention design, and statistical values that indicate the significant between-group effects. A positive outcome from the perspective of tailoring, for example, would be a statistically significant increase in self-reported fruit consumption, a bigger decrease in objectively measured weight, or a significant improvement in the stage of change of the intervention group compared with the control group.

**Table 2:** Objectives of behavior change, theories, intervention designs, and statistically significant outcomes of the tailored intervention groups compared with control groups

Study Authors and Year of Publication (n = 23)	Objectives of Behavior Change	Theories or Theoretical Concepts Mentioned	Intervention and Control Groups	Use of the Computer for Delivering Tailored Health Information	Statistically Significant Outcomes in Favor of Tailored Intervention Group Compared With Control Group^a^

Block et al, 2004 [53]	Nutrition	Transtheoretical Model or Stages of Change (TTM/SC)	Tailored fruit and vegetable consumption information Tailored fat information	Email	Change in fruit and vegetable consumption (all evaluation respondents)^d^ +0.73 times/day ^***^ Change in consumption of fat sources (all evaluation respondents)^d^ -0.39 times/day ^***^ Change in stage of change for fruit and vegetable consumption (all evaluation respondents)^d^^***^ Change in stage of change for fat (all evaluation respondents)^d^^***^
de Vet et al, 2008 [68]^b^	Nutrition	TTM/SC	Tailored precontemplation feedback Tailored contemplation feedback Tailored action feedback	Feedback-letter	-
Di Noia et al, 2008 [61]	Nutrition	TTM/SC, Concept of Self-efficacy (SE)	Tailored intervention General intervention	CD-ROM	Change in fruit and vegetable consumption was 38% higher for 1. vs 2., F_1,501_ = 26.62^***^ Change in pro (rather than con) phase of change^d^ F_1,501_ = 5.08 ^*^
Irvine et al, 2004 [57]	Nutrition	TTM/SC, SE, Theory of Reasoned Action (TRA)	Tailored intervention Waiting list control	Internet program	Change in fat consumption +0.24 vs +0.19 summary score points *t =* 8.44 ^**^ Change in fruit and vegetable consumption +0.36 vs +0.24 summary score points *t =* 6.49 ^***^ Change in stage of change to adopt a low fat diet +0.55 vs +0.50 summary score points *t =* 7.57 ^***^ Change in self-efficacy to decrease fat *t =* 3.87 ^***^

Kroeze et al, 2008 [21]^c^	Nutrition	TTM/SC	Tailored CD-ROM-delivered intervention Tailored print-delivered intervention General intervention	CD-ROM	1. vs 3. at 1 month Total fat intake^d^ 87.9(35.1) vs 104.2(44.1) g b = -10.93 ^*^ Saturated fat intake^d^ 32.8(15.2) vs 37.1(16.9) g b = -3.15 ^*^ Energy intake^d^ 9.1(3.0) vs 10.7(3.4) megajoules b = -1.07 ^*^
Luszczynska et al, 2007 [58]	Nutrition	SE	Tailored SE group Tailored SE + action planning group General intervention	Email	Change in fruit and vegetable consumption^d^ F_2,198_ = 6.81, η² = 0.07 ^***^
Oenema et al, 2001 [23]	Nutrition	SE, Precaution Adoption Model (PAPM)	Tailored intervention General intervention	Internet program	Change in awareness^d^ *t*_193_ = 3.82 ^***^ Change in intention to change diet^d^ *t*_195_ = 3.35 ^***^

Oenema et al, 2005 [15]^c^	Nutrition	PAPM, Elaboration Likelihood Model (ELM)	Tailored intervention General intervention No-information control	CD-ROM	Change in self-rated fat intake 1. vs 2. -0.13 vs +0.06 score points β = -0.10 ^*^ 1. vs 3. -0.13 vs +0.07 score points β = -0.10 ^**^ Change in self-rated vegetables intake 1. vs 2. -0.19 vs -0.07 score points β = 0.14 ^**^ 1. vs 3. -0.19 vs -0.05 score points β = 0.13 ^**^ Change in vegetable intake 1.vs 2. +0.1 vs -0.1 servings β = .08 ^*^ Change in intention to change (fat) 1. vs 2. +0.24 vs 0.00 score points β = -0.09 ^*^ 1. vs 3. +0.24 vs -0.03 score points β = -0.12 ^*^ Change in intention to change (vegetables) 1. vs 2. +0.34 vs +0.07 score points β = -0.13 ^*^ 1. vs 3. +0.34 vs +0.05 score points β = -0.14 ^**^
Papadaki and Scott, 2008 [62]	Nutrition	-	Tailored intervention General intervention	Email, Internet site	Change in vegetable intake +76.5 vs +27.7 g/d ^*^ Change in HDL (high-density lipoprotein) cholesterol +0.27 vs +0.07 mmol/l ^**^ Change in ratio of total:HDL cholesterol -0.47 vs -0.14 ^*^
Park et al, 2008 [63]^b^	Nutrition	TTM/SC, SE	Tailored intervention General intervention	Internet program	-

Dunton and Robertson, 2008 [54]	Physical activity	TTM/SC	Tailored intervention Waiting list control	Email, Internet site	Change in walking +69 vs +32 min/week β = 15.04(SE = 8.35) ^*^ Change in moderate to vigorous intensity physical activity +23 vs -25 min/week β = 17.02 (SE = 10.11) ^*^
Hageman et al, 2005 [56]^c^	Physical activity	SE, Health Promotion Model (HPM)	Tailored intervention General intervention	Newsletters	Change in cardiovascular fitness: VO² max^d^ F_1,26_ = 4.37 ^*^ Change in body fat %^d^ F_1,28_ = 6.46 ^*^
Marcus et al, 2007 [59]^b^	Physical activity	-	Tailored Internet-delivered interventions Tailored print-delivered intervention General intervention	Internet site	-
Napolitano et al, 2003 [60]	Physical activity	TTM/SC	Tailored intervention Waiting list control	Email, Internet site	Change in moderate to vigorous intensity physical activity at 1 month +29.5 vs +15.96 min/week F_1,54_ = 5.79 ^*^ Change in walking at 1 month +30.05 vs -3.78 min/week F_1,54_ = 12.1 ^***^ at 3 months +12.46 vs -15.4 min/week F_1,48_ = 5.2 ^*^
Spittaels et al, 2007 [65]^b^	Physical activity	TTM/SC, Theory of Planned Behavior (TPB)	Tailored advice + e-mails Tailored advice General advice	Email, Internet site	-
Spittaels et al, 2007 [72]	Physical activity	TTM/SC SE	Tailored advice + nontailored emails Tailored advice Waiting list control	Internet site	1. vs 2. vs 3. Change in active transportation 20 vs +24 vs +11 min/week F = 5.25 ^**^ Change in leisure-time physical activity +26 vs +19 vs -4 min/week F = 3.14 ^*^ Change in weekday sitting time -22 vs -34 vs +4 min/week F = 3.71 ^*^
Wanner et al, 2009 [70]^b^	Physical activity	TTM/SC SE	Tailored intervention General intervention Spontaneous users group	Email, Internet program	-
Frenn et al, 2005 [55]	Nutrition and physical activity	TTM/SC SE	Tailored intervention No-information control	Email, Internet site	Change in moderate to vigorous intensity physical activity +22 vs -46 min *t*_103_ = -1.99 ^*^ Change in dietary fat % -0.8 vs +0.1 g *t*_87_ = 2.73 ^**^
Oenema et al, 2008 [71]	Nutrition and physical activity	TTM/SC SE PAPM	Tailored intervention Waiting list control	Internet site	Change in saturated fat intake -1.61 vs -0.9 fat points b = -0.76 ^**^ Change in likelihood of meeting physical activity guidelines in the “at risk” group (low physical activity at baseline) +2.53 vs -0.45% odds ratio = 1.34, 95% confidence interval = 1.001-1.80 ^*^
Booth et al, 2008 [22]^b^	Weight management	TTM/SC, Goal Setting Theory, (GST)	Tailored advice + exercise Exercise only	Email, Internet site	-
Rothert et al, 2006 [64]	Weight management	SE	Tailored intervention General intervention	Internet program	Weight loss % 3(0.3) vs 1.2(0.4)% ^***^
Tate et al, 2001 [67]^c^	Weight management	-	Tailored intervention General intervention	Email	Weight loss 4.1(4.5) vs 1.6(3.3) kg *t =* 2.1 ^*^ Change in waist circumference 6.4(5.5) vs 3.1(4.4)cm ^**^
Tate et al, 2006 [66]^c^	Weight management	-	Computer-automated tailored counseling Human email counseling No counseling	Email, Internet program	1. vs 3. Weight loss at 3 months 5.3(4.2) vs -2.8(3.5) kg ^***^ Change in fat intake % at 6 months 37.3(6.6) vs 33.1(4.9) % ^**^

^a^ Statistical values presented are: mean (SD) (unless otherwise stated), F (F test, analysis of variance), *t* (*t* test), b (unstandardized regression coefficient), β (standardized regression coefficient), and η² **(**eta-squared**,** analysis of variance).

^b^ Only nonsignificant results were reported.

^c^ The effectiveness of the intervention is reported as mixed based on both significant and non-significant results.

^d^ Difference between baseline measurements and measurements at follow-up could not be calculated from presented data.

^*^ *P* ≤ .05

^**^ *P* ≤ .01

^***^ *P* ≤ .001

The intervention designs of 13 of the 23 studies included a tailored and a nontailored group, which received general, standard health information or feedback. Participants in the waiting list control groups of the 5 studies in which these were included received health information or feedback after the follow-up period, while 4 studies included a control group that did not receive health information or feedback even after follow-up (these were the no-information control groups, the no-counseling group, and the group receiving exercise only). In some studies, different delivery channels were also compared, for example, the Internet and print [59] or CD-ROM and print delivery [21]. Fourteen of the studies did not include a no-information control group [21,23,53,56,59,61-65,67,68,71].

The tailoring mechanism used in almost all of the studies was feedback. Studies in which participants were given more information were also able to use adaptation by matching the content to personal characteristics and needs. It must be noted that the tailoring mechanism applied was not always specified according to these terms. Personalization was mentioned in 2 studies [58,61].

The most often used channels for providing tailored feedback were Internet site (or Internet program), used in 15 of the 23 studies, and email, used in 11 studies. Moreover, various channels were utilized; for example, both email and Internet site were used in the study by Booth et al [22], while in other studies email and Internet sites were also combined with other media, such as video [55] or a diary and a peer support board [66]. The difference between Internet site and program was not always clear. In Table 2 these terms are used according to the term used in the original article.

In Table 2, only those outcomes are displayed that were statistically significant. Almost all studies, 21 of the 23, measured indicators connected to behavioral or physiological outcomes; the 2 that did not measured only psychosocial factors [23,63]. The majority of the studies (17) ended up with behavioral, physiological, or psychological between-group effects.

It is noteworthy that in 6 studies (2 on nutrition [63,68], 3 on physical activity [59,65,70], and 1 on weight management [22]), tailoring did not increase the effectiveness of the intervention, and consequently the overall outcome, from the perspective of tailoring, can be regarded as negative. By this we mean that some similar positive, neutral, or negative behavioral, physiological, or psychological outcomes were observed in both tailored and nontailored interventions. For example, no differences in self-reported and objectively measured physical activity were observed in either group over 13 months [70]. The results of the statistical analyses indicating nonsignificant outcomes are not presented in detail in Table 2.

Furthermore, it is noteworthy that in some studies the effectiveness of the intervention was reported as mixed from the perspective of tailoring [15,21,56,66,67]. This means that some measured indicators may have been better and others worse when compared with the control group. For example, Kroeze et al [21] reported that after one month both the Internet and print-delivered tailored intervention groups succeeded significantly better than the control group, but at three-month follow-up only the print-delivered tailored intervention group maintained a significant decrease in fat and dietary intake. In the case of weight loss, the same effect was reported by Tate et al [66]. Hageman et al [56], in turn, observed a significant between-group effect on secondary outcomes but not on the primary outcome, namely, physical activity.

Moreover, Tate et al [67] showed that the self-reported and objectively measured results might not always be in line. The tailored intervention group ended up with significantly greater objectively measured weight loss and greater reduction in waist circumference. However, participants in both groups reported changes in diet of similar magnitude despite significantly different magnitudes of weight loss.

Some of the studies attempted to measure psychosocial variables (such as intention, self-efficacy, and attitude toward the importance of diet) affecting the health behavior change or positive movement in the stage of change [15,23,53,57,58,61,63,70], but the variables were not always measured from the control groups or compared with their results. Moreover, it was shown that self-efficacy increased in the control group but decreased in the intervention group, and this was attributed to the fact that the intervention standard newsletters contained more motivational messages than the tailored ones [56]. In some studies, the immediate reaction to the tailored material was also examined. It was noted that the participants of the tailored intervention group reported more intention to change diet, appreciated tailored material more, and found tailored material more personally relevant [23,63-65].

### Possible Biases of the Second Generation Intervention Studies

When assessing outcomes, it is important to consider possible biases in the studies. For example, it must be noted that all studies relied on voluntary participants, which causes a self-selection bias. Moreover, the most common biases considered were: self-reporting as the only method of data collection, as in 15 of the 23 studies (see Table 1); lack of a pure no-information control group, as in 14 of the studies (see Table 2); overrepresentation of one sex even though the target group included both sexes, for example, more women than men, as in 10 of the studies [22,53,57-60,63,66,67,70], or more men than women, as in 2 of the studies [65,68].

Furthermore, in 10 of the studies, the participants differed from the national average in terms of their socioeconomic background (eg, education and income) [21,23,54,57,59,61,62,65,66,72], while in 3 of the studies, participants were more physically active than the national average [54,65,70]. In addition, in 3 studies the intervention situation was controlled [23,55,61], and in 2 studies the attrition rate was high [53,64].

In this content analysis, causalities were not investigated further. Thus the outcomes of the interventions from the perspective of tailoring were not examined in relation to the target group or the length of the study.

## Discussion

### Results and Implications for Research

Of the 23 studies selected, 10 focused on behavior change in nutrition, 7 on physical activity, 2 on nutrition and physical activity, and 4 on weight management. Most of the studies, 21 of 23, were randomized controlled trials. The target groups and the number of participants varied: 8 studies included more than 500 participants while 6 studies included less than 100. Most studies were short, that is, 6 months or less (20/23). Our analysis indicated that the outcomes of the studies were more positive regarding nutrition interventions, and it has been proposed that fruit and vegetable consumption is a relatively easy behavioral change to use as a first step [73]. However, the outcomes were less positive regarding physical activity interventions, as many studies ended up with negative outcomes from the perspective of tailoring (see Table 2). The physical activity measurements were conducted both objectively and by self-report. In 4 physical activity studies, the outcomes were mixed [56] or negative [59,65,70] from the perspective of tailoring. These results are in line with the studies by Neville et al [45] and Kroeze et al [30]. However, it must be noted that through our analysis we identified a bias effect in the intervention designs that may partly explain the differences in the outcomes of the physical activity interventions examined. Physical activity (as well as both physical activity and nutrition) interventions that did not end up with a significant between-group effect on physical activity measurements [56,59,65,70] used a general information control group, whereas those whose outcome was positive from the perspective of tailoring [54,55,60,71,72] had a no-information control group. Moreover, not all weight management interventions measuring physical activity [22,66,67] resulted in a significant effect on that parameter. This seems to be a result that needs more detailed analysis and empirical testing as, to the best of our knowledge, this has not previously been examined in detail.

Michie and Abraham [74] stated that “objective measures of behavior are likely to be the most informative outcomes when evaluating behavior change interventions.” The studies of this analysis used both objective measures and self-report. It must be noted that outcomes of self-report and objective measures of the same type of behavior do not always match, which was the case in two studies [67,70] included in our analysis. In the study by Wanner et al [70], self-reported changes in physical activity levels were not confirmed by objective measures. Tate et al [67] state that this was also the case in other studies. Participation itself may influence the perception of physical activity behavior and thus influence the levels of self-reported physical activity [70]. Moreover, it has been stated that “reported behavior change can also occur in the absence of actual behavior change due to social desirability effects” [74]. Therefore, the use of objective measures of physical activity may be important in determining whether the self-reported changes that are found are real [45].

Theories and models of health behavior change may help in understanding people’s decision-making and attitude changes, and extensive use of theory has been linked to increased intervention efficacy [38]. As in other studies [30], in our content analysis the TTM, including the stages of change, was the most popular theory mentioned. However, it must be noted that the TTM has been criticized, especially when applied for physical activity interventions [75], and has also supported with arguments emphasizing some promising results despite problems confronted in interventions [76].

To assess whether a tailored health behavior change program is effective, a long follow-up time of the intervention may be needed. As noted in other studies, in our analysis, too, 19 of the 23 studies were quite short in length, that is, 6 months or less. Although there is some evidence that even short-term interventions can be effective [8,44], they cannot be used as indicators of maintenance effect. Even though no change in the outcome was in evidence, it must be noted that an individual may feel that the program is personally relevant and this may foster attitudes toward health behavior change. In addition, the health effects of behavior change may also occur after many years. It has been proposed that estimates of health outcomes could be obtained using impact evaluations and epidemiological simulation models as an alternative to actual measurement [77]. 

The target groups varied widely, and specific minorities and risk groups were also studied. All studies relied on self-selected participants, whose high education level is one of the possible biases we have identified. Whether education level has an effect on attitudes and success in interventions has been under scrutiny. However, in one study, participants with low levels of education were even more positive than those with higher levels of education about how interesting and personally relevant they perceived tailored feedback to be [78]. This could be explained by the process of tailoring, which highlights only such information content that is perceived as the most relevant for the participant [79]. Therefore, tailoring can reduce the disadvantages associated with general health information on the Internet, namely, those related to incorrect information and also to incorrect understanding of the information content. Moreover, at the individual level, tailoring could be based on levels of information literacy, health literacy, and health information literacy (eg, [80] and [81]). These levels have not yet been widely applied in tailored interventions, though, some heuristics for tailoring materials to match the literacy levels have been presented as, for example, by Carstens [82].

It is quite new to apply tailoring in second generation interventions. In the selected interventions, several modes of delivery were used, such as email, Internet site and/or program, computer-delivered feedback letters, newsletters, and CD-ROM. Characteristics such as instantaneous feedback and appeal or engagement are potential advantages that new information and communication technologies (ICT) can provide and that may be of enormous benefit in achieving behavior change [83]. The third generation health communication emerges, and mobile devices are useful platforms for delivering health information. It has been claimed that “these platforms are also incorporating new functions such as sensing, monitoring, geospatial tracking, location based knowledge presentation, and host of other information processes that will potentially enhance the ability for accurate assessment and tailored feedback” [24]. Moreover, mobile devices can help to achieve “kairos,” that is, the opportune moment to persuade, and they can also be used for collecting self-reported data throughout everyday activities [24]. Combining second and third generation media, in this case the use of text messages, with other methods may prove successful, as it has been suggested that use of multiple methods of interaction with participants enhances the effectiveness of interventions [38].

### Implications for Practice

On the basis of this content analysis, the critical issues to be considered in planning and implementing a second generation tailored intervention study could be listed as follows: What health behavior change is the objective of the intervention? Does the intervention aim specifically at change in awareness, self-efficacy, motivation, or other factors influencing the behavior change as proposed by health behavior change theories and models? Will the intervention target one or multiple behaviors? What determinants affect the behavior selected and how can they be measured? What is the target group? What determinants of the target group must be taken into account (eg, cultural characteristics, health status, sociodemographic variables, knowledge, attitude, health information literacy)? Which tailoring mechanism is applied, and what is elicited in the assessment? What kind of an intervention design is applied? How is the intervention delivered? What is the length of the intervention? (For more information about the tailoring process, see [10,12,33,84].)

Moreover, biases, as identified in the studies, may have a significant effect on the outcomes of the intervention. Therefore, it is very important to consider how to minimize or even avoid biases. Related questions include: How do we get those at risk to participate in the study? How do we avoid self-selection bias? How can we activate men to participate? Could generating more technology oriented or third generation interventions make a difference in this? How do we get the most representative sample of the population? Should there be both a general information control and a no-information control group in order to achieve more reliable results?

### Strengths and Limitations of the Review

The strength of this review is its interdisciplinary approach. The number of selected articles was 23, which is in line with other meta-analyses. The goal of the content analysis was to find a sample of second generation intervention studies meeting the inclusion criteria. However, it must be noted that this is not a structured meta-analysis and cannot be replicated as such. On the other hand, we believe that the wide range of electronic databases searched may have helped us to find some studies that would have been missing in a structured meta-analysis. The number of references found by a literature search in Medline only would have been too high because the term “tailor” is used in many other ways, such as referring to the tailoring of medications or biochemical tests.

It is not easy to conduct a content analysis of intervention studies because methodological approaches, diversity of features, formats, channels for delivery, methods for providing feedback, goals, and ways to measure health behavioral changes differ greatly. Other authors have drawn the same conclusion, such as Lustria et al [8] and Abrams et al [85]. Likewise, researchers are many times forced to omit facts about technical factors or the details of tailoring from the articles, for example. The interventions selected for this content analysis were heterogeneous despite the strictly defined selection criteria. It is therefore demanding to develop generalized conclusions about the effectiveness of tailoring from such studies.

### Conclusion

At the individual level, behavior changes in nutrition, physical activity, and weight management can have a major role in preventing obesity, metabolic syndrome, and type 2 diabetes. This supports the individualist interpretation of behavioral strategies, which places emphasis on the responsibility of individuals for their health status and is supported by epidemiological studies.

To the best of our knowledge, this review is among the first to approach tailoring from this specific perspective in which second generation tailored intervention studies conducted in this context were analyzed. The 23 studies selected met the criteria for the content analysis of the specific aspects of the interventions: objectives of behavior change, target groups, sample sizes, lengths, attrition rates, theories applied, intervention designs, computer-based channels used, and the statistically significant outcomes of the interventions from the perspective of tailoring.

This review shows that the use of tailoring could have been effective in second generation interventions aimed at behavior change in nutrition, although the outcomes were mixed for physical activity and weight management. This conclusion is in line with earlier analyses. However, the analysis presented here adds to this knowledge by indicating the influence of biases on the outcomes of the interventions. In our analysis, the intervention design had a distinct effect on the outcomes of physical activity interventions. Thus, we suggest that the issue of bias should be considered more often in planning interventions and also considered in future meta-analyses. 

Tailoring of health information is the subject of research in various disciplines. It is one of the tools of persuasive technology, which aims to change attitudes or behaviors through persuasion and social influence [86,87]. An important aspect of interventions is information delivery. To accomplish this we must have an understanding of the information behavior and information practices of the people to whom the information to be delivered is tailored. The discipline of information studies has the potential to fill the gap in the existing knowledge and contribute to theory building within this multidisciplinary research area. This view is supported by the suggestion that information needs should be considered in tailoring [8].

## Abbreviations

ELM: Elaboration Likelihood Model
GST: Goal Setting Theory
HDL: high-density lipoprotein
HPM: Health Promotion Model
ICT: information and communication technologies
PAPM: Precaution Adoption Model
SE: concept of self-efficacy
TPB: Theory of Planned Behavior
TRA: Theory of Reasoned Action
TTM/SC: Transtheoretical Model or stages of change
